# A diagnostic classification version of Schizotypal Personality Questionnaire using diagnostic classification models

**DOI:** 10.1002/mpr.1807

**Published:** 2019-12-05

**Authors:** Chongqin Xi, Yan Cai, Siwei Peng, Jie Lian, Dongbo Tu

**Affiliations:** ^1^ School of Psychology Jiangxi Normal University Nanchang China

**Keywords:** diagnostic classification models (DCMs), G‐DINA model, schizotypal personality disorder (SPD), Schizotypal Personality Questionnaire (SPQ), symptom criteria‐level information

## Abstract

**Objective:**

To obtain more precise and rich information from the measurements for schizotypal personality disorder (SPD), a cutting‐edge psychometric theory called diagnostic classification models (DCMs) was first employed in the present study to develop a diagnostic classification version of the Schizotypal Personality Questionnaire (DC‐SPQ) based on the fifth edition of the *Diagnostic and Statistical Manual of Mental Disorders.*

**Methods:**

Under the framework of DCMs, 980 college students were recruited to calibrate item parameters of the Schizotypal Personality Questionnaire. Items that fit the psychometric characteristic would be selected to compose the DC‐SPQ, prior to an analysis of its indexes.

**Results:**

Results showed that the DC‐SPQ had high reliability and validity in both the classical test theory and DCMs, in addition to showing a sensitivity of 0.921 and a specificity of 0.841 with area under receiver operating characteristic curve = 0.936. Meanwhile, the four‐factor model proposed adequately fits with the data. More importantly, the DC‐SPQ provides not only the general‐level information similar to traditional questionnaires but also the symptom‐level information with the posterior probability, which provides an insight into delivering the individual‐specific intervention that is tailor made to schizotypal personality disorder.

**Conclusions:**

This study demonstrates that the DC‐SPQ is very valuable for psychometric detection in that it can clarify the symptom being measured and provide more reasonable estimates.

## 1 INTRODUCTION

Schizotypy commonly is considered as a latent psychological organization that putatively harbors the liability for schizophrenia, and it can manifest itself phenotypically variously, ranging from subclinical expression to full‐blown psychosis (Fonseca‐Pedrero & Debbané, 2017; Grant, Green, & Mason, 2018; Kwapil & Barrantes‐Vidal, 2015; Lenzenweger, 2018). As one of the manifestations or indicators of schizotypy, schizotypal personality disorder (SPD) is considered as a prototype characterized by impairments in identity, self‐direction, empathy, and/or intimacy, along with specific maladaptive traits in the domains of psychoticism and detachment (Fonseca‐Pedrero & Debbané, 2017; Kwapil & Barrantes‐Vidal, 2015; Lenzenweger, 2015). According to the fifth edition of the *Diagnostic and Statistical Manual of Mental Disorders* (DSM‐V; American Psychiatric Association, 2013), SPD is characterized by nine symptoms, including ideas of reference, social anxiety, odd/magical beliefs, unusual perceptions, odd/eccentric behavior, no close friends, odd speech, constricted affect, and suspiciousness. What is worth noting is that the nine symptom criteria of SPD set out by the DSM‐V are coherent with what is specified in the revision of the third edition of the *Diagnostic and Statistical Manual of Mental Disorders* (American Psychiatric Association, 1987).

Psychometric detection of individuals at risk for developing schizophrenia spectrum disorders is a critical enterprise. Currently, many of the self‐report measures of SPD have been developed for this purpose. Of these, several specific measurements that offer varying levels of, or focusing on specific, dimensions are widely used to conduct research into SPD, including the Schizotypal Personality Questionnaire (SPQ; Raine, 1991), the Wisconsin Schizotypy Scales (Winterstein et al., 2011), the Oxford–Liverpool Inventory of Feelings and Experiences (Mason, Claridge, & Jackson, 1995), the Five‐Factor Measure of Schizotypal Personality Traits (Edmundson, Lynam, Miller, Gore, & Widiger, 2011), and the Personality Diagnostic Questionnaire‐4 (PDQ‐4; Hyler, 1994). Among them, the SPQ (Raine, 1991), which mirrors nine schizotypal criteria of SPD that are laid out in the DSM‐V, is regarded as a very typical and most representative questionnaire of SPD, for which there has been a widespread application of it for the investigation into schizotypy from the clinical, neural, cognitive, and genetic aspects.

On the basis of the assumption that 10% of the population suffers with schizotypy (Lenzenweger, 2006; Lenzenweger & Korfine, 1992; Meehl, 1962), the top 10% of scorers on the SPQ sum score are categorized as having SPD (Raine, 1991). Despite this, there remain some drawbacks with this instrument. First, it is possible that the way of categorization that classes the top 10% scorers on the SPQ as having SPD is inappropriate for some particular populations, which is attributed to the fact that there is a wide range of disparities among different populations. Second, there is some ambiguity involved in the results provided by the SPQ for each respondent. Specifically, the SPQ is insufficient to illustrate further the structural difference of interindividual schizotypal personality feature in that subscale scores cannot confirm whether or not individuals possess those symptoms. Finally, the criteria for screening of the SPQ are different from those of the DSM‐V, which classes those who possess five or more symptom criteria as having SPD. To address these concerns, the present study employed diagnostic classification models (DCMs) to develop the diagnostic classification version of the SPQ (DC‐SPQ).

DCMs (Rupp, Templin, & Henson, 2010), also called cognitive diagnosis models (CDMs) by other researchers, are multidimensional categorical‐latent trait models that apply a set of categorical latent variables to characterize the observed response data. As compared with the classical test theory (CTT) and the item response theory (IRT), DCMs are deemed more appropriate under a circumstance where latent constructs are multidimensional and finer grained (Tu, Gao, Wang, & Cai, 2017). The ability of DCMs to make optimal use of the information in estimate and determine the interactions among attributes enables researchers to figure out to which extent a symptom described by an item will be observed given the different combinations of multiple disorders (de la Torre, van der Ark, & Rossi, 2015). Furthermore, DCMs can provide detailed estimation reports at the symptom level, whereas the CTT and IRT cannot. The most widespread application of DCMs is found in education with an aim to gain more understanding of students for their skills profile. As indicated by plenty of prior studies, however, DCMs have also been successfully applied to the measure of psychological disorder to identify the symptom profile for each patient (e.g., de la Torre et al., 2015; Jaeger, Tatsuoka, Berns, & Varadi, 2006; Templin & Henson, 2006; Tu et al., 2017).

Under the DCM framework, a DC‐SPQ was developed on the basis of the SPQ and DSM‐V in this study. Specifically, high‐quality items were selected to compose the DC‐SPQ after analyzing each item of the SPQ via appropriate DCMs. Then, the present study estimated the psychometric properties of the instrument developed, and examined its factorial structure. On the basis of the theoretic model predictions, we hypothesized that the DC‐SPQ has adequate psychometric properties. Furthermore, we further hypothesized that the proposed factor models of the SPQ still suited the DC‐SPQ. On the basis of the previous studies, we expected that three‐ or four‐factor models would provide a good fit to the data.

## 2 METHODS

### 2.1 Diagnostic criteria of SPD

Table 1 indicates the symptom criteria of SPD as defined in the DSM‐V, all of which have been examined by the SPQ. As specified by the DSM‐V, those who possess five or more symptom criteria will be defined as having SPD.

**Table 1:** Symptom criteria of Schizotypal Personality Disorder defined in the DSM‐V

ID	Symptom criteria
C1	Ideas of reference (excluding delusions of reference).
C2	Excessive social anxiety that does not diminish with familiarity and tends to be associated with paranoid fears rather than negative judgments about self.
C3	Odd beliefs or magical thinking that influences behavior and is inconsistent with subcultural norms (e.g., superstitiousness, belief in clairvoyance, telepathy, or “sixth sense”; in children and adolescents, bizarre fantasies or preoccupations).
C4	Unusual perceptual experiences, including bodily illusions.
C5	Behavior or appearance that is odd, eccentric, or peculiar.
C6	Lack of close friends or confidants other than first‐degree relatives.
C7	Odd thinking and speech (e.g., vague, circumstantial, metaphorical, overelaborate, or stereotyped).
C8	Inappropriate or constricted affect.
C9	Suspiciousness or paranoid ideation.

Abbreviation: DSM‐V, the fifth edition of the *Diagnostic and Statistical Manual of Mental Disorders.*

In DCMs, the parameters that a respondent has possessed each symptom are represented with the probability (i.e., the probability of possessing one symptom criterion), which can be converted in dichotomous scores (i.e., presence or absence) by comparing them to a cut score (usually 0.5; de la Torre, Hong, & Deng, 2010; García, Olea, & De la Torre, 2014; Templin & Henson, 2006). These parameters would be calculated to estimate each respondent's symptom profile and the posterior probability of SPD (PP‐SPD) according to the DSM‐V, that is to say the PP‐SPD represents the posterior probability of possessing five or more symptoms of SPD defined in the DSM‐V. Therefore, the formulation of the PP‐SPD can be expressed as

(1) $$\mathrm{PP}‐{\mathrm{SPD}}_{i}=\sum_{l=1}^{L}\prod_{k=1}^{K}\left[P_{\mathrm{ik}}^{\mathrm{lk}}\cdot {\left(1-P_{\mathrm{ik}}\right)}^{1-\mathrm{lk}}\right],$$

where *PP* ‐ *SPD*_*i*_ is the PP‐SPD for individual *i* according to the DSM‐V; *L* represents all symptom profiles, which have five or more symptoms, and thus, there are $\left({}_{5}^{9}\right)+\left({}_{6}^{9}\right)+\left({}_{7}^{9}\right)+\left({}_{8}^{9}\right)+\left({}_{9}^{9}\right)=256$ symptom profiles; *lk* = 1 represented possessing symptom *k* in the *l* symptom profile and 0 if it did not; and *P*_*ik*_ is the probability that individual *i* possesses symptom *k*, and *P*_*ik*_ can be estimated by DCMs. In the analysis, the respondents scoring over 0.5 for PP‐SPD are classed as having the potential to suffer with SPD.

### 2.2 Diagnostic classification models

Under the DCM framework, the attribute in education setting situations commonly is treated as latent variables with two statuses, mastery or nonmastery, such as skills, cognitive processes, or solution strategies. As far as mental disorders are concerned, however, the attribute is typically replaced by a symptom or disorder (de la Torre et al., 2015). The symptom profiles are usually referred to as attribute patterns or latent classes (*K* attributes will yield 2^*K*^ attribute patterns) and are denoted by *α*_*l*_ = (*α*_*l*1_,  …, *α*_*lk*_,  …,  *α*_*lK*_), where *α*_*lk*_ = 1 if the respondents in the latent class *l* have satisfied symptom criterion *k* and 0 if they have not. For instance, symptom vector *α*_*l*_(11010) indicates that respondents in latent class *l* have possessed symptoms 1, 2, and 4 but have not symptoms 3 and 5 (García, Olea, & De la Torre, 2014). Besides, for *l* = 1 to *L*, where *L* represents the number of all possible combinations of the symptoms. For item *j*, the endorsement probability is subjected only to the influence of the measured symptom, and the symptom profiles measured by item *j* is denoted as $\alpha_{\mathrm{lj}}^{*}=\left(\alpha_{l1},\;\ldots ,\;\alpha_{\mathrm{lk}},\;\ldots ,\;\alpha_{{\mathrm{lK}}_{j}^{*}}\right)$, where $K_{j}^{*}=\sum_{k=1}^{K}q_{\mathrm{jk}}$ represents the number of required symptoms measured for item *j.* The DCMs are aimed at associating respondents' item responses with their symptom profiles.

In DCMs, a fundamental component is the so‐called Q‐matrix (Tatsuoka, 1983), which indicates the symptoms measured by each item. A Q‐matrix is typically constructed on the basis of the opinions of domain experts, clinical theories, or the results from empirical research (de la Torre et al., 2015). The Q‐matrix is a *J* (number of items) × *K* (number of symptoms) binary matrix, where entry 1 signifies that a symptom is measured by an item and entry 0 signifies it is not. For example, *q*_*jk*_ = 1 if symptom *k* is measured by item *j* and 0 otherwise.

A number of DCMs that range in generality have been advanced in recent years (e.g., de la Torre, 2009; de la Torre et al., 2015; Henson, Templin, & Willse, 2009; Junker & Sijtsma, 2001; von Davier, 2008), such as the generalized deterministic input, noisy, “and” gate (G‐DINA; de la Torre, 2011) model, the loglinear diagnostic classification model (LDCM; Henson et al., 2009), and the general diagnostic model (GDM; von Davier, 2008). These general DCMs are also referred to as saturated models, considering all possible interactions among symptoms. Meanwhile, some models, which are commonly called as reduced models, have been proposed for the assumption that a symptom interacts in some particular manner, such as deterministic inputs, noisy, “and” gate (DINA; Junker & Sijtsma, 2001) and deterministic input, noisy, “or” gate (DINO; Templin & Henson, 2006). It is to be noted that many of existing DCMs are regarded as a special case of the G‐DINA model.

The formulation of the G‐DINA model (de la Torre, 2011) is expressed as

(2) $$P\left(X_{j}=1|\alpha_{\mathrm{lj}}^{*}\right)=\delta_{j0}+\sum_{k=1}^{K_{j}^{*}}\delta_{\mathrm{jk}}\alpha_{\mathrm{lk}}+\sum_{k^{'}=k+1}^{K_{j}^{*}}\sum_{k=1}^{K_{j}^{*}-1}\delta_{{\mathrm{jkk}}^{'}}\alpha_{\mathrm{lk}}\alpha_{{\mathrm{lk}}_{\ldots }^{'}}+\delta_{j12\ldots k_{j}^{*}}\prod_{k=1}^{K_{j}^{*}}\alpha_{\mathrm{lk}},$$

where $P\left(X_{j}=1|\alpha_{\mathrm{lj}}^{*}\right)$ is the endorsement probability that a respondent from latent class *l* has symptom profile $\alpha_{\mathrm{lj}}^{*}$; *δ*_*j*0_ is the intercept for item *j*, which represents the baseline probability; *δ*_*jk*_ is the main effect due to possessing symptom *k*; $\delta_{{\mathrm{jkk}}^{'}}$ is the interaction effect due to possessing symptoms *k* and *k*^′^; and $\delta_{j12\ldots k_{j}^{*}}$ is the interaction effect due to possessing all symptoms measured by item *j.*

Considering an item measuring two attributes, the corresponding formula of the G‐DINA can be expressed as

(3) $$P\left(X_{j}=1|\alpha_{\mathrm{lj}}^{*}\right)=\delta_{j0}+\delta_{j1}\alpha_{l1}+\delta_{j2}\alpha_{l2}+\delta_{j12}\alpha_{l1}\alpha_{l2},$$

where *δ*_*j*0_ is the baseline probability, which is the probability of an endorsement for respondents when none of the required symptoms is present; *δ*_*j*1_ and *δ*_*j*2_ are the main effects of symptoms *α*_*l*1_ and *α*_*l*2_, respectively, which indicate the increase in probability of an endorsement for respondents who possess symptoms *α*_*l*1_ and *α*_*l*2_, respectively; and *δ*_*j*12_ is the interaction effect, which is the change in the probability of a correct response due to possession of both symptoms *α*_*l*1_ and *α*_*l*2_.

Currently, as a saturated CDM, the G‐DINA model is one of the most representative of DCMs in that many existing DCMs are the special case of it with some constraints or hypotheses (de la Torre, 2011; Tu et al., 2017). The DINA model is a special case of the G‐DINA model by setting all the parameters, except *δ*_*j*0_ and $\delta_{j12\ldots k_{j}^{*}}$, to zero; ACDM also is a special case of the G‐DINA model by supposing no interaction effects, namely there is only the parameters of intercept δ_j0_ and main effect δ_jk_, and so on. In addition, the G‐DINA model is also widely used in educational and psychological research (e.g., de la Torre et al., 2015; Jaeger et al., 2006; Templin & Henson, 2006; Tu et al., 2017). The parameters of the G‐DINA model can be estimated through using the marginal maximum likelihood estimation algorithm.

### 2.3 Instruments

#### 2.3.1 The SPQ

The SPQ includes a total of 74 items with a binary answer of “yes” or “no,” with 1 point assigned to the response of “yes.” As for the reliability, the coefficients of Cronbach's alpha and test–retest correlation were 0.91 and 0.82 for the total measure, respectively (Raine, 1991). In the level of the subscale, the coefficients of Cronbach's alpha range from 0.81 (odd beliefs) to 0.63 (constricted effect; Raine, 1991). With respect to the validity, among the top 10% of scorers on the SPQ, as many as 55% of them were diagnosed with an SPD (Raine, 1991). Besides, the nine subscales and the total scale held respectively significant positive correlations with the scores of the Structured Clinical Interview for DSM‐III‐R Personality (SCID‐II; Spitzer et al., 1987) (*r* = .68, *p* < .001; Raine, 1991). The SPQ has been demonstrated to have adequate psychometric properties in many studies (e.g., Chen et al., 1997; Cicero, 2015; Fonseca‐Pedrero et al., 2014; Raine, 1991). Furthermore, a cross‐national study, conducted by Fonseca‐Pedrero et al. (2018) using a large and multinational sample, also first suggested that the SPQ has good psychometric properties across countries. In order to compose a DC‐SPQ, all of the items were selected from the Chinese‐translated version of the SPQ (Chen et al., 1997) based on the psychometric characteristic.

As mentioned by Templin and Henson (2006), what plays an essential role in the validity of the diagnostic results for a cognitive diagnostic research lies in the establishment of the Q‐matrix. In this study, the work that specifies which symptom is needed to respond to each item had been accomplished by Raine (1991). The Q‐matrix contains nine columns, one for each of the nine criteria. As shown in Table 2, the Q‐matrix indicates that each item only measures one symptom criterion, and each symptom criterion was measured by an average of 8.2 items.

**Table 2:** Q‐matrix for the part items of SPQ

Item	Symptom criterion of schizotypal personality disorder								
	C1	C2	C3	C4	C5	C6	C7	C8	C9
1	0	1	0	0	0	0	0	0	0
2	0	0	0	0	1	0	0	0	0
3	0	0	0	0	0	0	1	0	0
4	0	0	0	0	0	0	0	0	1
5	1	0	0	0	0	0	0	0	0
6	0	1	0	0	0	0	0	0	0

*Note.* Criteria C1 to C9 represent respectively the nine symptom criteria for schizotypal personality disorder defined in the DSM‐V in Table 1. Element 1 in row *j* and column *k* in the Q‐matrix presents that symptom *k* was measured by item *j*, and element 0 presents that item *j* does not measure symptom *k.*

Abbreviations: DSM‐V, the fifth edition of the *Diagnostic and Statistical Manual of Mental Disorders*; SPQ, Schizotypal Personality Questionnaire.

Some item examples in the SPQ are shown in Table 3, where “I sometimes avoid going to places where there will be many people because I will get anxious” measures “Excessive social anxiety that does not diminish with familiarity and tends to be associated with paranoid fears rather than negative judgments about self” (C2), whereas item “Have you had experiences with the supernatural? “ measures “Odd beliefs or magical thinking that influences behavior and is inconsistent with subcultural norms (e.g., superstitiousness, belief in clairvoyance, telepathy, or ‘sixth sense'; in children and adolescents, bizarre fantasies or preoccupations)” (C3).

**Table 3:** Some item examples in the SPQ

Items	Q‐matrix								
	C1	C2	C3	C4	C5	C6	C7	C8	C9
I sometimes avoid going to places where there will be many people because I will get anxious.	0	1	0	0	0	0	0	0	0
Have you had experiences with the supernatural?	0	0	1	0	0	0	0	0	0
Have you often mistaken objects or shadows for people, or noises for voices?	0	0	0	1	0	0	0	0	0
People sometimes find it hard to understand what I am saying.	0	0	0	0	0	0	1	0	0
People sometimes find me aloof and distant.	0	0	0	0	0	0	0	1	0

*Note.* Criteria C1 to C9 represent respectively the nine symptom criteria for schizotypal personality disorder defined in the DSM‐V in Table 1. Element 1 in row *j* and column *k* in the Q‐matrix presents that symptom *k* was measured by item *j*, and element 0 presents that item *j* does not measure symptom *k.*

Abbreviations: DSM‐V, the fifth edition of the *Diagnostic and Statistical Manual of Mental Disorders*; SPQ, Schizotypal Personality Questionnaire.

#### 2.3.2 The PDQ‐4

The PDQ‐4 (Hyler, 1994), which aims to measure the 10 specific personality disorders defined in the DSM‐IV (American Psychiatric Association, 2000), was employed to examine the validity of the DC‐SPQ in this study. For the subscale such as Schizotypal, 9 is the maximum possible subscale score, and people who score over 5 for Schizotypal are classed as having the potential to suffer with SPD. However, plenty of studies suggested that the way of categorization of the Schizotypal subscale for Chinese college students reached a minimum of 6 points rather than 5 points (Fu, 2004; Fu, Yao, & Yu, 2008; Li, 2006; Lin, Meng‐Cheng, Mu‐Li, & Xiong‐Zhao, 2013). Currently, many studies indicated that the PDQ‐4 had suitable psychometric properties in both the initial version (Hyler, 1994) and the various cultures version. In this study, the Chinese‐translated version of the PDQ‐4 (Yang et al., 2000) was applied to examine the validity of the DC‐SPQ.

### 2.4 Participants

A total of 1,362 Chinese college students were recruited from seven colleges in three cities of China in this study. To protect personal privacy, each respondent was assigned a unique code number for identification. This survey consists of the basic demographic questions, the SPQ, and the exclusion criteria. To screen out individuals who randomly responded, three lie detection items that were designed as opposite meanings according to three SPQ items were embedded in this survey. For an original item of the SPQ such as “Do you sometimes get concerned that friends or coworkers are not really loyal or trustworthy?” its corresponding lie detection item was “I find it easy to trust other people.” Participants who responded to any one of the three pair items using the same answer were eliminated in this study.

Finally, 1,116 respondents participated and completed the pencil‐and‐paper tests. Of those, 5.1% (*n* = 57) participants were eliminated due to lie detection items, and 1.4% (*n* = 16) participants also were excluded due to meeting any preestablished exclusion criteria shown as follows: (a) prior diagnosis of physical diseases; (b) prior diagnosis of organic brain diseases as a result of infection, trauma, tumor, genes, or vascular diseases; (c) prior diagnosis of cognitive impairment or amentia; (d) prior diagnosis of depression or other mental disorders caused by alcohol or drug addiction; or (e) having greatly affected by some event in the previous 1 month (Yunfei, Zeping, & Zhen, 2006; Rui, 2012). In addition, there were 63 (5.6%) partial completers, and most of the missing values appeared in the three variables of age, family locus, and gender. Therefore, the MissMech R package (Jamshidian, Jalal, & Jansen, 2014) was employed to test the assumption that data are missing completely at random (Rubin, 1976). Given the result of the test supporting the assumption of missing completely at random (Rubin, 1976) and a low percentage of missing data (5.6% and <10%; Bennett, 2001), missing values were eliminated using the listwise deletion method, resulting in a final sample of 980 (87.8%) participants.

The age of the sample ranges from 16 to 25, with the mean value of 20.5 (*SD* = 1.79). In this study, all participants are of Chinese ethnicity, and 62.3% (*n* = 611) are female. Besides, 43.1% (*n* = 422) of the sample come from an urban area, and 25.8% (*n* = 253) are an only child. As for the grade, the distribution was as follows: 50.6% (*n* = 496) freshman, 18.9% (*n* = 185) sophomore, 17.4% (*n* = 171) junior, 11.3% (*n* = 111) senior, and 1.7% (*n* = 17) master. The item parameters of the SPQ were calibrated by the responses of all these participants via DCMs.

To examine the sensitivity and specificity of the DC‐SPQ, 685 individuals from the current sample were recruited as a validation sample to fill out the pencil‐and‐paper tests of the Schizotypal subscale from the PDQ‐4 (Hyler, 1994). The validation sample consisted of a healthy control group (*N*_1_ = 609) and an SPD high‐risk group (*N*_2_ = 76). The SPD high‐risk group represents a class of people whose Schizotypal score from the PDQ‐4 (Hyler, 1994) reached a minimum of 6 points, with the rest being grouped into the healthy control group.

### 2.5 Statistical analysis

#### 2.5.1 DCM for the SPQ

Currently, although many DCMs have been developed, it remains unclear which one is the most appropriate model for a given data set. Commonly, the most appropriate model for each item was selected through the Wald test, and the reduced model can substitute the saturated model via the Wald test in some cases (de la Torre, 2011; Ma, Iaconangelo, & de la Torre, 2016). However, all models are mathematically equivalent when an item only measures one symptom. Given that (a) all items of the SPQ only measure one symptom criterion and (b) the G‐DINA model is deemed as the most representative and fairly typical of DCMs, the G‐DINA model was employed in this article.

#### 2.5.2 Analyzing the psychometric characteristics for each item and choosing the high‐quality item to compose the DC‐SPQ

First, the psychometric characteristics were analyzed for each item using the G‐DINA model. These psychometric characteristics include *S‐* *X*^2^ item fit statistic (Orlando & Thissen, 2000, 2003), differential item functioning (DIF; e.g., female and male) detected by the Wald test statistic (Hou, De la Torre, & Nandakumar, 2014), and the discrimination as expressed in Formula (4) (de la Torre, 2008). Following an analysis of the psychometric characteristics for each item of the SPQ, items that are compliant with the following criteria would be excluded: low discrimination items (<0.30), DIF items (*p* < .01), and items with poor item fit (*p* < .01); then the remaining items were selected to comprise the DC‐SPQ. In this article, the CDM R package (Robitzsch, Kiefer, & George, 2018) was applied to estimate the *S‐* *X*^2^ item fit statistic, DIF, and discrimination index (*Disc*_*j*_), which is defined as

(4) $${\text{Disc}}_{j}=P\left(X_{j}=1|\alpha_{\mathrm{lj}}^{*}=1\right)-P\left(X_{j}=1|\alpha_{\mathrm{lj}}^{*}=0\right),$$

where $P\left(X_{j}=1|\alpha_{\mathrm{lj}}^{*}=1\right)$ is the probability of item endorsement for respondents who have possessed all the symptoms measured by item *j* and $P\left(X_{j}=1|\alpha_{\mathrm{lj}}^{*}=0\right)$ is the probability of item endorsement for the respondents who have not possessed any symptom measured by item *j.* Therefore, *Disc*_*j*_ was defined as the difference in the probabilities of item endorsement of item *j* between examinees who possess all required symptoms of item *j* and those who do not (de la Torre, 2008).

#### 2.5.3 The analysis of reliability and validity for the DC‐SPQ

In this study, the reliability coefficients of Guttman split‐half and McDonald's omega (ω; McDonald, 1999) in the full scale and the McDonald's omega in the subscale were both calculated on the basis of the CTT. Under the DCM framework, the classification consistency reliability (Cui, Gierl, & Chang, 2012) of the nine symptoms was also estimated using the CDM R package (Robitzsch et al., 2018). On the basis of the validation sample, criterion‐related validity and convergent validity were estimated by computing the correlation between the DC‐SPQ and the Schizotypal subscale from the PDQ‐4 (Hyler, 1994). Furthermore, cross‐validation was conducted using the healthy control group and SPD high‐risk group to examine the validity of the DC‐SPQ further. Specifically, multivariate analysis of covariance (MANCOVA) was conducted to test group differences (healthy control group vs. SPD high‐risk group) on the DC‐SPQ scores and PP‐SPD (with gender and family locus as the covariates). In addition, sensitivity, specificity, and screening odds ratio of the DC‐SPQ were also estimated. Sensitivity is the probability of screening positive (by the DC‐SPQ) if the respondent was an SPD high‐risk individual (according to the PDQ‐4). Specificity is the probability of screening negative (by the DC‐SPQ) if the respondent was a healthy individual (according to the PDQ‐4). There was greater power to distinguish high‐risk individuals or healthy individuals as long as the larger value of sensitivity or specificity is known.

#### 2.5.4 Structure estimation

In this article, the unidimensional sum scores of items would represent the symptom scores for each of the nine DSM‐V criteria. Confirmatory factor analysis was estimated using the maximum likelihood estimation via Mplus 7 to test existent four typical models of the SPQ, namely, the unidimensional model, three‐factor model (Raine et al., 1994), four‐factor model (Stefanis et al., 2004), and bifactor model (Preti et al., 2015). Several fit indexes were calculated to assess model fit: the ratio of *x*^2^/*df*, comparative fit index (CFI), Tucker–Lewis index (TLI), root mean square error of approximation (RMSEA), and standardized root mean square residual (SRMR). CFI and TLI values of 0.90 or higher, *x*^2^/*df* values of 3.0 or lower, RMSEA values of 0.08 or lower, and SRMR values of 0.09 or lower are considered acceptable (Marsh, Hau, & Grayson, 2005).

#### 2.5.5 Screening score reporting

The class proportions for each symptom estimated by the DC‐SPQ (using DCMs) and PDQ‐4 (using CTT) would be calculated. Also, the Kappa test was conducted to investigate the interrater agreement between the two methods in each symptom. Qualitative Kappa‐value agreement descriptions were 0.81–1.0 = “almost perfect,” 0.61–0.80 = “substantial,” 0.41–0.60 = “moderate,” 0.21–0.40 = “fair,” and 0.0–0.20 = “slight” (Landis & Koch, 1977). On the other hand, the nine symptom classification correlation was also calculated in this study.

## 3 RESULTS

### 3.1 Item analysis of the DC‐SPQ

Forty‐seven high‐quality items, as shown in Table 4, were finally selected to comprise the DC‐SPQ according to three statistical indexes (i.e., discrimination, item fit, and DIF). The discrimination index of selected items, ranging from 0.302 to 0.569 with an average of 0.415, is fairly indicative of a high discrimination demonstrated by all the items of the DC‐SPQ. Under a circumstance where the significance level is α = .01, the DC‐SPQ has good item fit with no DIF discovered between female and male. Besides, the number of items measuring the nine symptoms varies from 3 to 7 with an average of 5.2. The entire item content of the DC‐SPQ and its Q‐matrix are shown in Table A1 **.**

**Table 4:** The psychometric characteristics of items selected in the DC‐SPQ

Number of items	Discrimination	Model fit			DIF (female and male)		
		*S‐* *X*^2^	*df*	*p*	Wald stat.	*df*	*p*
1	.34	39.58	43	.62	2.13	2	.35
2	.38	46.05	43	.35	1.49	2	.48
3	.49	36.26	43	.76	3.22	2	.20
4	.31	44.97	43	.39	1.33	2	.52
5	.41	38.86	43	.65	6.20	2	.05
6	.40	62.87	43	.03	0.06	2	.97
7	.32	37.32	43	.72	0.73	2	.70
8	.33	38.16	43	.68	0.30	2	.86
9	.50	47.59	43	.29	5.47	2	.07
10	.30	37.94	43	.69	0.15	2	.93
11	.54	33.91	43	.84	8.13	2	.02
12	.32	30.25	43	.93	7.95	2	.02
13	.37	38.64	43	.66	7.18	2	.03
14	.53	60.22	43	.04	5.29	2	.07
15	.31	41.83	43	.52	4.90	2	.09
16	.35	28.23	43	.96	1.74	2	.42
17	.43	52.84	43	.14	4.88	2	.09
18	.50	39.13	43	.64	1.80	2	.41
19	.40	32.30	43	.88	7.93	2	.02
20	.40	52.22	43	.16	4.87	2	.09
21	.55	61.34	43	.03	5.00	2	.08
22	.57	40.39	43	.59	6.31	2	.04
23	.57	69.06	43	.01	4.73	2	.09
24	.48	32.88	43	.87	0.91	2	.63
25	.40	49.02	43	.25	0.08	2	.96
26	.35	53.64	43	.13	2.97	2	.23
27	.36	46.92	43	.32	1.42	2	.49
28	.33	53.53	43	.13	0.17	2	.92
29	.43	53.85	43	.12	1.60	2	.45
30	.32	47.30	43	.30	2.19	2	.33
31	.46	49.72	43	.22	1.60	2	.45
32	.44	43.34	43	.46	0.20	2	.90
33	.30	44.60	43	.41	4.84	2	.09
34	.36	55.58	43	.10	1.68	2	.43
35	.39	38.17	43	.68	1.27	2	.53
36	.31	49.61	43	.23	5.03	2	.08
37	.39	34.78	43	.81	1.23	2	.54
38	.56	42.51	43	.49	1.97	2	.37
39	.49	40.11	43	.60	11.32	2	.01
40	.39	36.59	43	.74	7.05	2	.03
41	.41	43.30	43	.46	0.66	2	.72
42	.43	48.15	43	.27	7.03	2	.03
43	.45	34.08	43	.83	0.26	2	.88
44	.49	66.77	43	.01	5.53	2	.06
45	.42	49.31	43	.24	0.61	2	.74
46	.51	34.85	43	.81	0.99	2	.61
47	.39	38.94	43	.65	2.00	2	.37

Abbreviations: DC‐SPQ, diagnostic classification version of the Schizotypal Personality Questionnaire; DIF, differential item functioning.

### 3.2 Reliability and validity

Under the CTT, the reliability coefficients of the full‐scale Guttman split‐half and McDonald's omega were 0.857 and 0.867, respectively. Besides, nine McDonald's omega coefficients in the subscales were, in decreasing order, 0.695 (odd/eccentric behavior), 0.670 (odd speech), 0.628 (suspiciousness), 0.615 (constricted effect), 0.580 (no close friends), 0.579 (ideas of reference), 0.570 (social anxiety), 0.534 (unusual perceptions), and 0.347 (odd/magical beliefs). On the other hand, the range of classification consistency reliability of the nine symptoms based on DCMs is from 0.783 to 0.914 with an average of 0.874. The above results indicate that the results from DCMs could improve reliability coefficients for a subscale or symptom, because it is easier to classify it into one of two groups in the DCM framework, but the CTT framework needs to locate the true score. As for the validity, an excellent content validity is illustrated by the results of the DC‐SPQ measuring all nine symptoms of SPD defined in the DSM‐V. On the other hand, the DC‐SPQ also showed good convergent validity as the PP‐SPD based on DCMs has a significant correlation of 0.687 (*p* < .001) with the Schizotypal score of the PDQ‐4 (Hyler, 1994). All of these results indicate that the DC‐SPQ has good reliability and validity based on both the CTT and DCMs.

**Figure 1:**
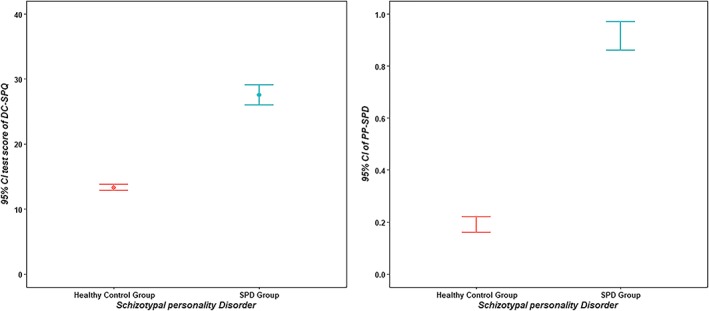
Error bar graph of the DC‐SPQ scores and PP‐SPD for two groups. DCMs, diagnostic classification models; DC‐SPQ, diagnostic classification version of the Schizotypal Personality Questionnaire; DSM‐V, the fifth edition of the *Diagnostic and Statistical Manual of Mental Disorders*; PP‐SPD, the posterior probability of schizotypal personality disorder, which was calculated based on the DC‐SPQ and the diagnostic criteria in the DSM‐V via DCMs; 95% CI, 95% confidence interval

Additionally, cross‐validation was conducted for the healthy control group (*N*_1_ = 609) and the SPD high‐risk group (*N*_2_ = 76). As shown in Figure 1, the error bar indicates that the two groups have clearly different DC‐SPQ scores and PP‐SPD, with reasonably symmetric distributions. On the other hand, group differences (healthy control group vs. SPD high‐risk group) for the DC‐SPQ scores and PP‐SPD were estimated with gender and family locus as covariates. Box's *M* statistic indicated that there was a significant difference between the groups in the assumption of variance–covariance matrix homogeneity, Box's *M* = 29.681, *F*(3, 211,560) = 9.796, *p* < .001. The MANCOVA showed a significant main effect, *Pillai's Trace* = .332, *F*(2, 680) = 169.233, *p* < .001, partial *η*^2^ = .332, and gender (*p* < .01) was a significant covariate, but family locus (*p* = .446) was not a significant covariate in this study. On the basis of the results of MANCOVA, there was a significant difference between the groups on the DC‐SPQ scores, *F*(1, 681) = 301.759, *p* < .001, partial *η*^2^ = .307, and PP‐SPD, *F*(1, 681) = 292.185, *p* < .001, partial *η*^2^ = .300. In particular, the SPD high‐risk group was significantly higher than the healthy control group in terms of both the DC‐SPQ scores and PP‐SPD.

On the basis of the healthy control group and the SPD high‐risk group, the sensitivity and specificity of the DC‐SPQ were respectively 0.921 and 0.841 using the 50% PP‐SPD via DCMs, and the area under receiver operating characteristic curve was 0.936. The result above indicated that the probability of SPD high‐risk individuals (according to the PDQ‐4) being screened positive by the DC‐SPQ is 92.1%, and the probability of healthy individuals (according to the PDQ‐4) being screened negative by the DC‐SPQ is 84.1%. On the other hand, the odds ratio of screening is 60.833 (*p* < .001) with a 95% confidence interval of [25.709, 143.946]. It is illustrated by the result that the DC‐SPQ can effectively distinguish SPD high‐risk individuals from healthy individuals.

### 3.3 Structure estimation

**Figure 2:**
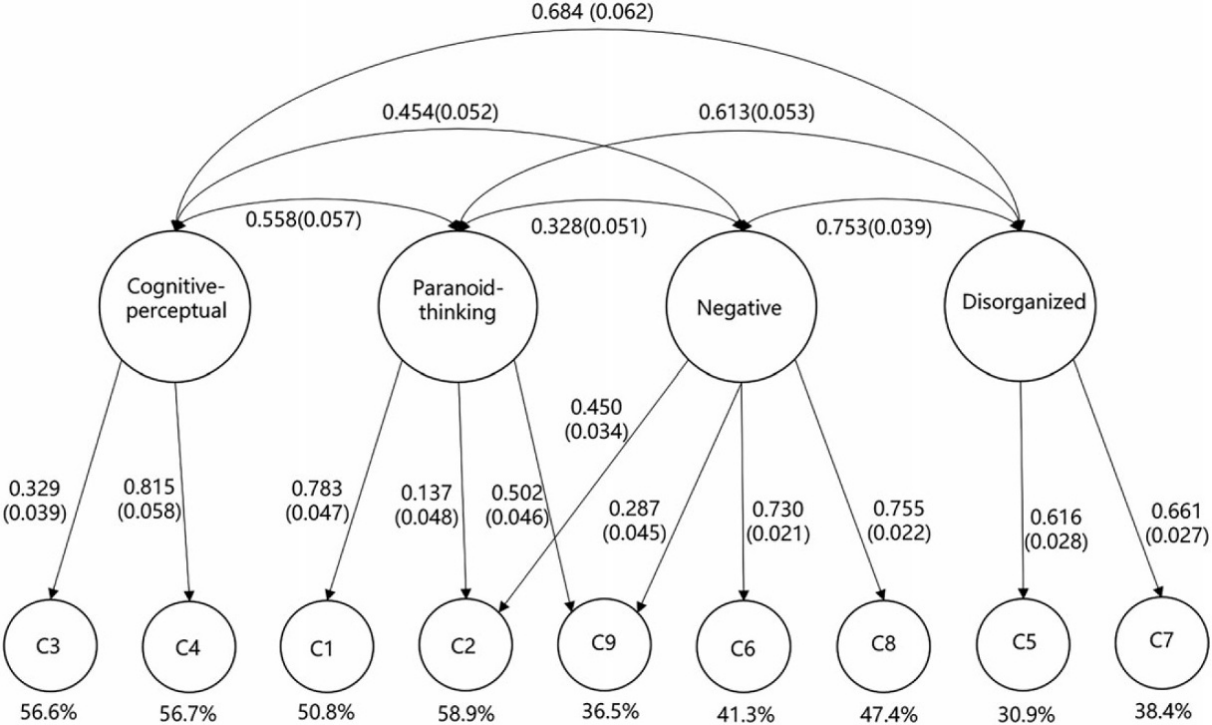
Factor structure with standardized factor loadings. C, criterion

The fit indices of the four models proposed are shown in Table 5. As depicted, the four‐factor model (Stefanis et al., 2004) provides the best fit with the real data: $\;x_{\left(19\right)}^{2}$ = 129.29, CFI = 0.94, TLI = 0.90, RMSEA = 0.07, and SRMR = 0.03. Although the *x*^2^ value was significant, there is no surprise according to the sample size and model complexities of this study (Callaway, Cohen, Matthews, & Dinzeo, 2014; Kline, 2005). Besides, the other fit indices were all found to be within the desired ranges. On the other hand, the standardized factor loadings and the factor correlation indices of the DC‐SPQ four‐factor model were reported in Figure 2. The estimates for all factor loading of this study were significantly different from 0, with most of them greater than the critical value 0.30 (Wen, 2008). The results of the structure estimation provided by this study are considerably similar to those found by Stefanis et al. (2004).

**Table 5:** Fit indexes for confirmatory factor analysis

Model	***x***^**2**^	*df*	***x***^**2**^/*df*	RMSEA	CFI	TLI	SRMR
Unidimensional model	383.52	27	14.20	0.12	0.82	0.76	0.06
Three‐factor model (Raine et al., 1994)	185.819	23	8.08	0.09	0.92	0.87	0.04
Bifactor model (Preti et al., 2015)	156.163	18	8.68	0.09	0.93	0.86	0.04
Four‐factor model (Stefanis et al., 2004)	129.29	19	6.80	0.07	0.94	0.90	0.03

Abbreviations: CFI, comparative fit index; RMSEA, root mean square error of approximation; SRMR, standardized root mean square residual; TLI, Tucker–Lewis index.

### 3.4 Screening score reporting

The ultimate score reports provided by this article are not only the posterior probability that each respondent satisfied at least five of the symptoms defined in the DSM‐V but also the detailed symptom‐level information. Table 6 showed the class proportions for each symptom estimated by the DC‐SPQ (using DCMs) and PDQ‐4 (using the CTT), as well as the results of the Kappa test, which investigated the interrater agreement between the two methods in each symptom. Results of the Kappa test indicated substantial agreement for symptom 8 (Kappa value = 0.64); moderate agreement for Symptom 9 (Kappa value = 0.54) and Symptom 2 (Kappa value = 0.43); fair agreement for Symptom 5 (Kappa‐value = 0.38), Symptom 4 (Kappa value = 0.37), Symptom 7 (Kappa value = 0.30), and Symptom 1 (Kappa value = 0.22); and slight agreement for Symptom 6 (Kappa value = 0.19) and Symptom 3 (Kappa value = 0.16). On the other hand, Table 7 indicated that there were significant correlations between all nine symptoms except for a pair of symptoms (Symptoms 3 and 8). Specifically, the correlation coefficient between Symptoms 3 and 2, Symptoms 3 and 6, and Symptoms 3 and 7 were all less than 0.20, which indicated that Symptom 3 has clear distinction with those symptoms. In addition, the correlation coefficient between Symptoms 6 and 8 and Symptoms 4 and 9 were all greater than 0.65, which showed that the two pairs of symptoms have relatively high correlation. The remaining symptom classification correlations were between the above two coefficients, and most of them are less than 0.5.

**Table 6:** The proportions of possessing each symptom

Scales and its' Kappa‐test	C1	C2	C3	C4	C5	C6	C7	C8	C9
DC‐SPQ	0.32	0.43	0.41	0.31	0.27	0.34	0.36	0.48	0.31
PDQ‐4	0.39	0.32	0.36	0.31	0.14	0.12	0.16	0.43	0.29
*κ*‐coefficient	0.22***	0.43***	0.16***	0.37***	0.38***	0.19***	0.30***	0.64***	0.54***

*Note.* Criteria C1 to C9 represent respectively the nine symptom criteria for schizotypal personality disorder defined in the DSM‐V in Table 1.

Abbreviations: DC‐SPQ, diagnostic classification version of the Schizotypal Personality Questionnaire; DSM‐V, the fifth edition of the *Diagnostic and Statistical Manual of Mental Disorders*; PDQ‐4, Personality Diagnostic Questionnaire‐4.

*** *p <* .001.

**Table 7:** Nine symptom classification correlation by the DC‐SPQ

Criterion or Symptom	C1	C2	C3	C4	C5	C6	C7	C8	C9
C1	1								
C2	.391^**^	1							
C3	.436^**^	.147^**^	1						
C4	.573^**^	.368^**^	.525^**^	1					
C5	.506^**^	.237^**^	.363^**^	.618^**^	1				
C6	.250^**^	.474^**^	.172^**^	.501^**^	.584^**^	1			
C7	.429^**^	.414^**^	.105^**^	.441^**^	.509^**^	.498^**^	1		
C8	.235^**^	.585^**^	.043	.393^**^	.339^**^	.679^**^	.588^**^	1	
C9	.559^**^	.294^**^	.415^**^	.659^**^	.621^**^	.546^**^	.462^**^	.428^**^	1

*Note.* Criteria C1 to C9 represent respectively the nine symptom criteria for schizotypal personality disorder defined in the DSM‐V in Table 1.

Abbreviations: DC‐SPQ, diagnostic classification version of the Schizotypal Personality Questionnaire; DSM‐V, the fifth edition of the *Diagnostic and Statistical Manual of Mental Disorders.*

*** *p <* .001.

**Figure 3:**
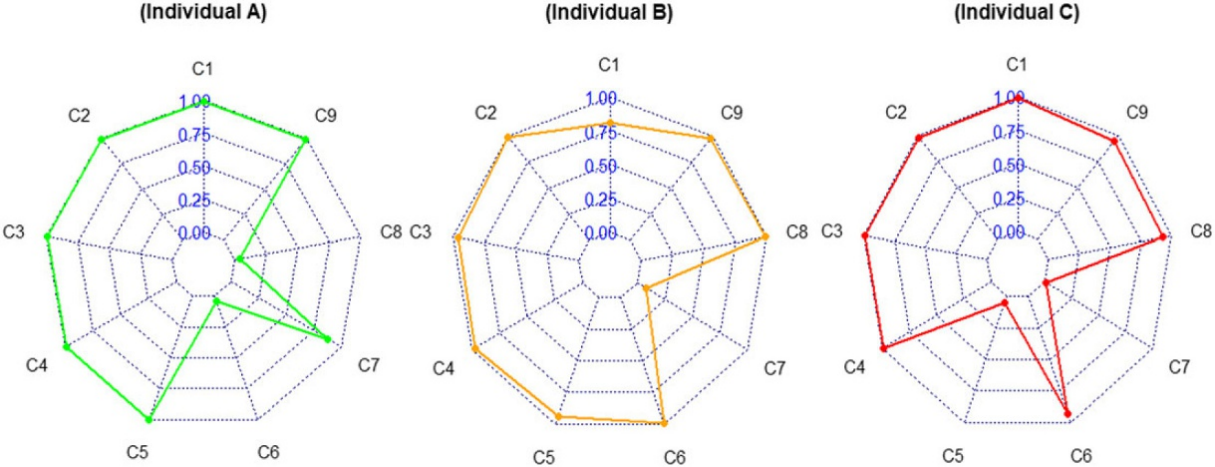
Symptom spectrum of schizotypal personality disorder for three individuals. C1–C9 represent the nine symptom criteria for schizotypal personality disorder in the fifth edition of the *Diagnostic and Statistical Manual of Mental Disorders* in Table 1

In order to illustrate the unique information produced by DCMs, detailed score reports for three respondents were provided as an example in that they got the same Schizotypal score from the PDQ‐4 (Hyler, 1994) and were being classed as SPD high‐risk individuals by the PDQ‐4 (Hyler, 1994). Table 8 and Figure 3 show the PP‐SPD and symptom spectrum for the three individuals, respectively.

**Table 8:** Individual example estimates

Individual	Symptom criterion									PP‐SPD
	C1	C2	C3	C4	C5	C6	C7	C8	C9	
A	0.99	0.99	0.99	0.99	1.00	0.04	0.87	0.04	0.99	0.99
B	0.81	0.99	0.96	0.97	0.94	0.99	0.08	0.99	0.98	0.99
C	0.99	0.98	0.99	0.99	0.05	0.93	0.01	0.93	0.95	0.99

*Note.* Criteria C1 to C9 represent respectively the nine symptom criteria for schizotypal personality disorder defined in the DSM‐V in Table 1.

Abbreviations: DCMs, diagnostic classification models; DC‐SPQ, diagnostic classification version of the Schizotypal Personality Questionnaire; DSM‐V, the fifth edition of the *Diagnostic and Statistical Manual of Mental Disorders*; PP‐SPD, the posterior probability of schizotypal personality disorder, which was calculated based on the DC‐SPQ and the diagnostic criteria in the DSM‐V via DCMs.

As shown in Table 8, due to the same PP‐SPD values of 0.99, the three individuals were all classed as SPD high‐risk individuals by the DC‐SPQ, which is in line with the screening result of the PDQ‐4. Despite the three individuals A, B, and C having the same screening results in the general level, there remain disparities at the symptom criteria level. From Table 8 and Figure 3, Individual A (female, 20 years old, a freshman of college, and from the countryside) probably meets seven symptoms except for Criteria 6 and 8; Individual B (male, 21 years old, a junior of college and from the city) probably meets eight symptoms except for Criterion 7; and Individual C (female, 21 years old, a junior of college, and from the city) probably meets seven symptoms except for Criteria 5 and 7. This detailed symptom‐level information gives insight for tailoring individual‐specific diagnosis and treatments for SPD; it would contribute to increasing the treatment's effectiveness.

## 4 DISCUSSION

The outstanding contribution of this study is to develop a DC‐SPQ using DCMs based on the DSM‐V and SPQ for the first time. Specifically, the G‐DINA model was employed to analyze the psychometric characteristics of each item in the SPQ, and then high‐quality items were chosen to compose the DC‐SPQ on the basis of these psychometric characteristics. Subsequently, this study estimated the psychometric properties of the DC‐SPQ and provided a detail screening score report for each respondent. The results of this article indicate that the DC‐SPQ not only has good reliability and validity in both CTT and DCMs but also shows sufficient goodness of fit between the four‐factor model (Stefanis et al., 2004) and the real data. Also, the DC‐SPQ can effectively distinguish between the healthy individuals and the SPD high‐risk individuals in the two groups classed by the PDQ‐4.

Aside from that, the DC‐SPQ is capable of providing more reasonable and rich information for each respondent. First, different from the traditional approach of detection that requires researchers to select the top 10% scorers on the SPQ in different populations, the DC‐SPQ could facilitate a highly efficient and reasonable assessment to be conducted as the screening approach that is consistent with the DSM‐V. Second, the screening result of the DC‐SPQ was provided with the PP‐SPD, which is effective in gaining us a comprehensive understanding of the psychometric case identification. Third, the probability of possessing one symptom criterion is very valuable for further research into the structural difference of interindividual schizotypal personality features as well as interventional purposes.

There remain some limitations on this study despite the promising results that have already been obtained. Foremost, the sample involved in this study consists of 980 college students, which may not represent the entire population. Miettunen et al. (2010) indicated that the education level might influence self‐reported schizotypy; that is, younger adults commonly report higher levels of symptoms. This phenomenon also was verified in Chinese college students via the PDQ‐4 (e.g., Fu, 2004; Fu et al., 2008; Li, 2006; Lin et al., 2013), which explains the reason why the way of categorization of the Schizotypal subscale reached a minimum of 6 points rather than 5 points in this study. However, borrowing Templin and Henson's (2006) idea, these differences make up an important part of the ongoing process of understanding the SPD and are not necessarily detrimental to the analysis of this article. Second, the present study did not collect information on the IQ or socioeconomic status of the samples, which may be more appropriately used as covariables than may gender and family locus. Third, the limits of self‐report measurements used in this study also are considered in that they only provide information on subjective‐perceived attitudes. Therefore, there is inherent inadequacy while classing SPD via self‐report rather than the clinical diagnosis conducted by clinical professionals. Fourth, under the CTT framework, although the DC‐SPQ has desirable reliability in the full scale, there is poor Omega reliability in the subscale of the odd/magical beliefs due to few items. In summary, further validation is still required, which is to come not only from those studies using a large sample size with the entire sample having rich information on socio‐demographic characteristics but also from clinical diagnosis stemmed from the trained clinical professionals.

Besides, the method used in this study is more complicated than are the existing methods (such as CTT and IRT), which would hamper its application in practice. Therefore, user‐friendly software that provides clear guidelines for the interpretation of screening results is essential in the future (de la Torre et al., 2015). Finally, the DC‐SPQ remains possible to add to the burden on a patient as it consists of 47 items. Although Moore, Calkins, Reise, Gur, and Gur (2018) have developed the computerized adaptive testing version of the SPQ to provide much fewer items but equal valid assessment, it remains incapable of providing the symptom‐level information for patients. Further studies could explore the application of the computerized adaptive test to the DC‐SPQ to conduct more reasonable assessment but with much fewer items.

## CONFLICT OF INTEREST

The authors declare that the research was conducted in the absence of any commercial or financial relationships that could be construed as a potential conflict of interest.

## FUNDING INFORMATION

This study was funded by the National Natural Science Foundation of China (31660278, 31960186, and 31760288).

## THE ENTIRE ITEM CONTENT OF THE DC‐SPQ AND ITS Q‐MATRIX

The entire item content of the DC‐SPQ, which is marked (e.g., *), and its Q‐matrix are shown in Table A1. Criteria C1 to C9 represent respectively the nine symptom criteria for schizotypal personality disorder defined in the DSM‐V. Element 1 in row *j* and column *k* in the Q‐matrix presents that symptom *k* was measured by item *j*, and element 0 presents that item *j* does not measure symptom *k.*

**Table A1:** The SPQ and its entire initial Q‐matrix

No.	Item	Q‐matrix								
		C1	C2	C3	C4	C5	C6	C7	C8	C9
1	Do you sometimes feel that things you see on the TV or read in the newspaper have a special meaning for you?	1	0	0	0	0	0	0	0	0
2*	I sometimes avoid going to places where there will be many people because I will get anxious.	0	1	0	0	0	0	0	0	0
3	Have you had experiences with the supernatural?	0	0	1	0	0	0	0	0	0
4	Have you often mistaken objects or shadows for people, or noises for voices?	0	0	0	1	0	0	0	0	0
5*	Other people see me as slightly eccentric (odd).	0	0	0	0	1	0	0	0	0
6	I have little interest in getting to know other people.	0	0	0	0	0	1	0	0	0
7*	People sometimes find it hard to understand what I am saying.	0	0	0	0	0	0	1	0	0
8	People sometimes find me aloof and distant.	0	0	0	0	0	0	0	1	0
9*	I am sure I am being talked about behind my back.	0	0	0	0	0	0	0	0	1
10*	I am aware that people notice me when I go out for a meal or to see a film.	1	0	0	0	0	0	0	0	0
11*	I get very nervous when I have to make polite conversation.	0	1	0	0	0	0	0	0	0
12	Do you believe in telepathy (mind reading)?	0	0	1	0	0	0	0	0	0
13*	Have you ever had the sense that some person or force is around you, even though you cannot see anyone?	0	0	0	1	0	0	0	0	0
14	People sometimes comment on my unusual mannerisms and habits.	0	0	0	0	1	0	0	0	0
15*	I prefer to keep myself to myself.	0	0	0	0	0	1	0	0	0
16	I sometimes jump quickly from one topic to another when speaking.	0	0	0	0	0	0	1	0	0
17*	I am not good at expressing my true feelings by the way I talk and look.	0	0	0	0	0	0	0	1	0
18	Do you often feel that other people have it in for you?	0	0	0	0	0	0	0	0	1
19*	Do some people drop hints about you or say things with a double meaning?	1	0	0	0	0	0	0	0	0
20	Do you ever get nervous when someone is walking behind you?	0	1	0	0	0	0	0	0	0
21	Are you sometimes sure that other people can tell what you are thinking?	0	0	1	0	0	0	0	0	0
22	When you look at a person, or yourself in a mirror, have you ever seen the face change right before your eyes?	0	0	0	1	0	0	0	0	0
23*	Sometimes other people think that I am a little strange.	0	0	0	0	1	0	0	0	0
24*	I am mostly quiet when with other people.	0	0	0	0	0	1	0	0	0
25	I sometimes forget what I am trying to say.	0	0	0	0	0	0	1	0	0
26	I rarely laugh and smile.	0	0	0	0	0	0	0	1	0
27*	Do you sometimes get concerned that friends or coworkers are not really loyal or trustworthy?	0	0	0	0	0	0	0	0	1
28	Have you ever noticed a common event or object that seemed to be a special sign for you?	1	0	0	0	0	0	0	0	0
29*	I get anxious when meeting people for the first time.	0	1	0	0	0	0	0	0	0
30*	Do you believe in clairvoyance (psychic forces, fortune telling)?	0	0	1	0	0	0	0	0	0
31*	I often hear a voice speaking my thoughts aloud.	0	0	0	1	0	0	0	0	0
32*	Some people think that I am a very bizarre person.	0	0	0	0	1	0	0	0	0
33*	I find it hard to be emotionally close to other people.	0	0	0	0	0	1	0	0	0
34	I often ramble on too much when speaking.	0	0	0	0	0	0	1	0	0
35*	My “nonverbal” communication (smiling and nodding during a conversation) is not very good.	0	0	0	0	0	0	0	1	0
36*	I feel I have to be on my guard even with friends.	0	0	0	0	0	0	0	0	1
37	Do you sometimes see special meanings in advertisements, shop windows, or in the way things are arranged around you?	1	0	0	0	0	0	0	0	0
38*	Do you often feel nervous when you are in a group of unfamiliar people?	0	1	0	0	0	0	0	0	0
39	Can other people feel your feelings when they are not there?	0	0	1	0	0	0	0	0	0
40	Have you ever seen things invisible to other people?	0	0	0	1	0	0	0	0	0
41	Do you feel that there is no one you are really close to outside of your immediate family, or people you can confide in or talk to about personal problems?	0	0	0	0	0	1	0	0	0
42*	Some people find me a bit vague and elusive during a conversation.	0	0	0	0	0	0	1	0	0
43*	I am poor at returning social courtesies and gestures.	0	0	0	0	0	0	0	1	0
44*	Do you often pick up hidden threats or put‐downs from what people say or do?	0	0	0	0	0	0	0	0	1
45*	When shopping do you get the feeling that other people are taking notice of you?	1	0	0	0	0	0	0	0	0
46	I feel very uncomfortable in social situations involving unfamiliar people.	0	1	0	0	0	0	0	0	0
47*	Have you had experiences with astrology, seeing the future, UFOs, ESP, or a sixth sense?	0	0	1	0	0	0	0	0	0
48*	Do everyday things seem unusually large or small?	0	0	0	1	0	0	0	0	0
49	Writing letters to friends is more trouble than it is worth.	0	0	0	0	0	1	0	0	0
50*	I sometimes use words in unusual ways.	0	0	0	0	0	0	1	0	0
51*	I tend to avoid eye contact when conversing with others.	0	0	0	0	0	0	0	1	0
52*	Have you found that it is best not to let other people know too much about you?	0	0	0	0	0	0	0	0	1
53*	When you see people talking to each other, do you often wonder if they are talking about you?	1	0	0	0	0	0	0	0	0
54	I would feel very anxious if I had to give a speech in front of a large group of people.	0	1	0	0	0	0	0	0	0
55*	Have you ever felt that you are communicating with another person telepathically (by mind reading)?	0	0	1	0	0	0	0	0	0
56*	Does your sense of smell sometimes become unusually strong?	0	0	0	1	0	0	0	0	0
57*	I tend to keep in the background on social occasions.	0	0	0	0	0	1	0	0	0
58*	Do you tend to wander off the topic when having a conversation?	0	0	0	0	0	0	1	0	0
59*	I often feel that others have it in for me	0	0	0	0	0	0	0	0	1
60	Do you sometimes feel that other people are watching you?	1	0	0	0	0	0	0	0	0
61*	Do you ever suddenly feel distracted by distant sounds that you are not normally aware of?	0	0	0	1	0	0	0	0	0
62	I attach little importance to having close friends.	0	0	0	0	0	1	0	0	0
63*	Do you sometimes feel that other people are talking about you?	1	0	0	0	0	0	0	0	0
64*	Are your thoughts sometimes so strong that you can almost hear them?	0	0	0	1	0	0	0	0	0
65*	Do you often have to keep an eye out to stop people from taking advantage of you?	0	0	0	0	0	0	0	0	1
66*	Do you feel that you cannot get “close” to people?	0	0	0	0	0	1	0	0	0
67*	I am an odd, unusual person.	0	0	0	0	1	0	0	0	0
68*	I do not have an expressive and lively way of speaking.	0	0	0	0	0	0	0	1	0
69*	I find it hard to communicate clearly what I want to say to people.	0	0	0	0	0	0	1	0	0
70*	I have some eccentric (odd) habits.	0	0	0	0	1	0	0	0	0
71	I feel very uneasy talking to people I do not know well	0	1	0	0	0	0	0	0	0
72*	People occasionally comment that my conversation is confusing.	0	0	0	0	0	0	1	0	0
73*	I tend to keep my feelings to myself.	0	0	0	0	0	0	0	1	0
74	People sometimes stare at me because of my odd appearance.	0	0	0	0	1	0	0	0	0
